# Editorial: The intersection of gene regulation and metabolism in cardiovascular disease

**DOI:** 10.3389/fgene.2023.1253690

**Published:** 2023-08-28

**Authors:** Julie Pires Da Silva, Pamela A. Padilla, Anastacia M. Garcia

**Affiliations:** ^1^ Institut des Maladies Métaboliques et Cardiovasculaires, I2MC, Université de Toulouse, Inserm, Université Toulouse III—Paul Sabatier (UPS), UMR1297, Toulouse, France; ^2^ Department of Biological Sciences, University of North Texas, Denton, TX, United States; ^3^ Division of Cardiology, Department of Pediatrics, Children’s Hospital Colorado, University of Colorado Anschutz Medical Campus, Aurora, CO, United States

**Keywords:** cardiovascular disease, genetics, epigenetics, risk factors, personalized medicine, cardiac metabolism

## Introduction

**FIGURE 1:**
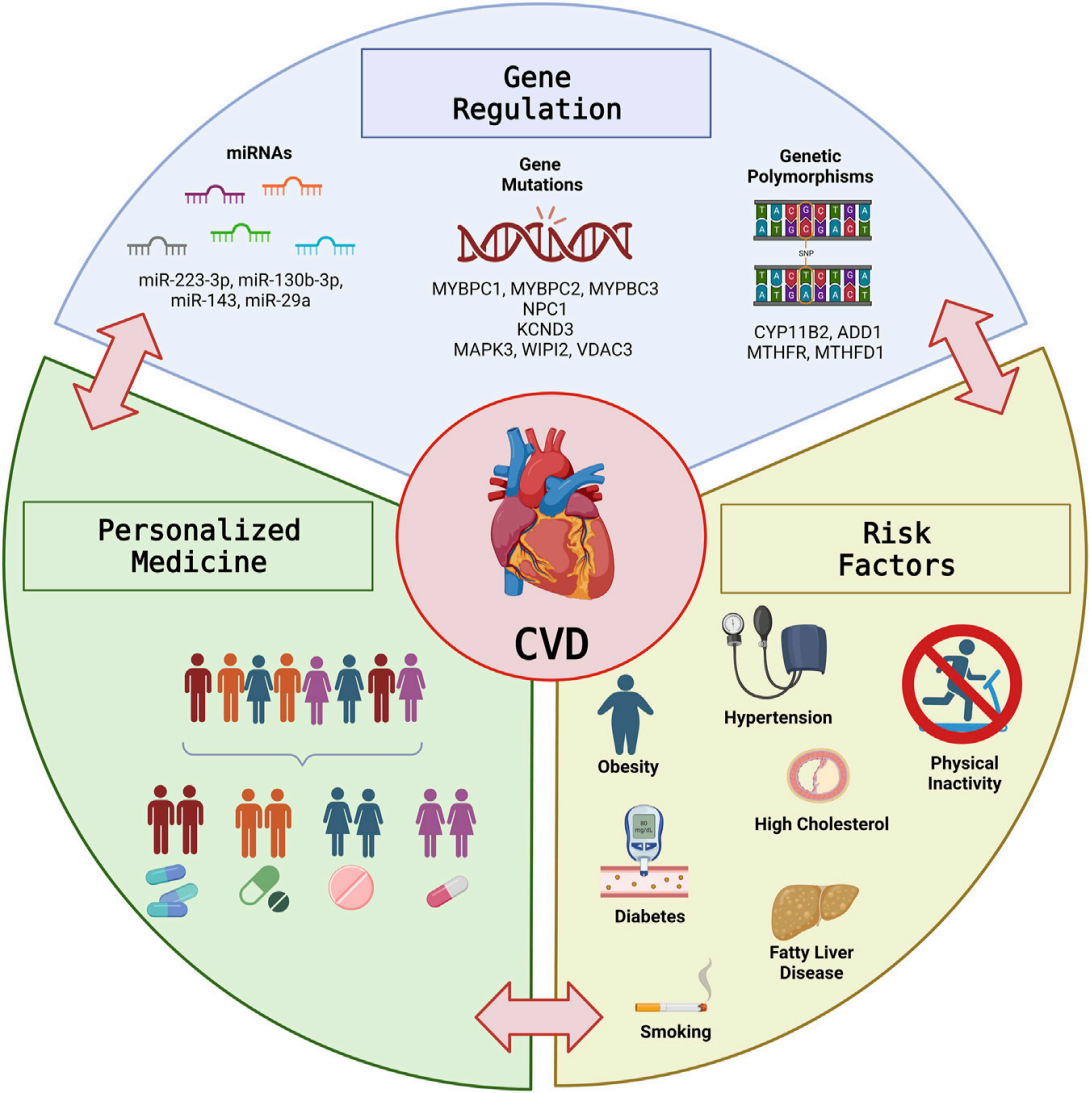
The Intersection of Gene Regulation and Metabolism in Cardiovascular Disease. Graphical illustration of the intersection between Gene regulation and metabolic risk factors in CVD and the potential for personalized medicine to optimize treatment strategies and improve outcomes in the growing CVD population. This figure was prepared with BioRender.

Cardiovascular disease (CVD) is the leading cause of death globally, accounting for more than 30% of all deaths worldwide. Numerous studies have demonstrated that the various etiologies of CVD are complex and multiple genetic and environmental components likely contribute to the observed clinical manifestations. Here, we outline recent advances and challenges related to the intersection of genetics, gene regulation, and metabolic dysfunction in CVD, and the promises pharmacogenetics and personalized medicine offer to optimize CVD treatment strategies and improve outcomes in this growing population (Figure 1).

## Genetic mutations contributing to CVD

Complex cardiovascular diseases stemming from genetic mutations have become a worldwide concern. Mutations in myosin binding protein-C (MyBP-C), a sarcomeric protein which regulates the force of contraction in striated muscles, for example, can present as a variety of cardiac and skeletal myopathies. Leveraging a machine learning approach Desai et al., systematically assess the evolutionarily conserved and epigenetic patterns of MYBPC family mutations. The highest prevalence of coding variants and pathogenic coding variants was observed in cardiac MYBPC3. The cardiac specific N-terminal domain is highly prone to variations, thus may be crucial for the development of treatments for hypertrophic cardiomyopathy. Moreover, a stark change in the polarity of amino acids was observed in missense mutations, potentially altering protein binding and/or key post-translational modifications in MyBP-C proteins, thereby leading to a disease phenotype. Together, these results can be used for genetic mapping and identification of genetic variants in individuals with a history of MYBPC mutations for the purpose of clinical diagnosis and prognosis.

Sudden cardiac death (SCD) accounts for 4-5 million deaths globally and is responsible for more than 50% of all cardiovascular deaths. Rare genetic variants have been recently shown to contribute to important causes of SCD. Zhao et al., identified a novel indel variation of the Niemann-Pick type C1 (NPC1) gene, a regulator of cholesterol homeostasis, that is associated with increased susceptibility of SCD in Chinese populations. This novel indel may serve as a useful genetic marker for SCD risk stratification as well as molecular diagnosis. Moreover, mutations of genes encoding ion channels often display mixed electrophysiological phenotypes and lead to a rare disease called cardiocerebral channelopathy, which significantly increases the risk of SCD. Zhang H et al. report a case study of an 11-year-old girl with cardiocerebral channelopathy. Using whole-exome sequencing, a Potassium Voltage-Gated Channel Subfamily D Member 3 (KCND3) mutation was identified. The novel KCND3 mutation in this patient was shown to be the primary cause of disease, highlighting the growing importance of next-generation sequencing in precision medicine.

Acute myocardial infarction (AMI) is also a leading cause of cardiovascular-related death and disability worldwide. Ferroptosis, an iron-dependent programmed cell death, is a major driver of ischemic injury in the heart. Huang et al., assessed the efficiency of ferroptosis-related genes (FRGs) for early diagnosis of AMI, and utilized a machine learning approach to construct a prediction model for AMI, that was validated in two independent cohorts. The results suggest that key ferroptosis-related markers (MAPK3, WIPI2 and VDAC3) are involved in the progression of AMI, providing a new direction for early diagnosis and potential molecular targets for optimal treatment of AMI.

## CVD risk factors and personalized medicine

Due to the rise in prevalence of major risk factors CVD, such as high blood pressure, physical inactivity, diabetes mellitus and obesity, the burden of CVD is expected to worsen in the decades to come. Lodewijks et al., discuss the role of molecular crosstalk between adipose tissue and the heart, with a focus on endocrine and paracrine signaling, immune cells, inflammatory cytokines, and inter-organ communication through non-coding RNAs. This review highlights that interception of specific disease-causing signals between adipose tissue and the myocardium could provide a novel therapeutic axis for intervention and improved outcomes.

Moreover, genetic polymorphisms are a major cause of interindividual differences in the presentation of CVD risk factors and response to therapy. Zhang P et al. detected genetic polymorphisms of aldosterone synthase (CYP11B2) and alpha-adducing (ADD1), two genes playing key roles in essential hypertension, a primary risk factor of CVD. The identification of specific polymorphisms will allow for better detection of CVD risk factors leading to personalized medicine approaches that have the potential to improve outcomes in the long-term. Similarly, hyperhomocysteinemia (HHcy) is characterized by a high level of homocysteine and is yet another risk factor for CVD and brain-related disorders. Folate supplementation is the common treatment, but genetic polymorphism of key enzymes of one-carbon metabolism such as methylenetetrahydrofolate reductase (MTHFR) or formyltetrahydrofolate synthetase 1 (MTHFD1) interact with its efficacy. Liu et al. sequenced the genome of a family with hereditary HHcy, and identified a doubly bi-allelic variant in the MTHFR and MTHFD1 genes with a novel variant in the MTHFR gene and an already well-known homozygous variant in the MTHFD1 gene. Folic acid treatment was therefore not sufficient for this patient, further highlighting an important role for whole-exome sequencing as a tool for personalized medicine.

Lastly, while the relationship between non-alcoholic fatty liver disease (NAFLD) and CVD remains unclear, Chew et al., discuss the genetic studies of NAFLD overlapping with CVD, and describe both the colinear and opposing correlations of genetic associations between the two . This review therefore suggests increased understanding of the genetic cross-talk between NAFLD and CVD will shed light on important modulators of these disease states and has the power to improve polygenic risk scores for more accurate risk prediction and personalized prevention and treatment strategies.

## Conclusion

Together, the studies published in this Research Topic highlight multiple genetic and environmental components that contribute to the clinical manifestations of CVD. Increased identification of additional novel disease-associated mutations and increased understanding of the complex interactions between genetics, environmental risk factors, and metabolism, are necessary for the identification of additional ways to improve diagnosis, risk prediction, and personalized treatment strategies for cardiovascular and cardiometabolic disease.

